# Development and Validation of the Body Size Scale for Assessing Body Weight Perception in African Populations

**DOI:** 10.1371/journal.pone.0138983

**Published:** 2015-11-04

**Authors:** Emmanuel Cohen, Jonathan Y. Bernard, Amandine Ponty, Amadou Ndao, Norbert Amougou, Rihlat Saïd-Mohamed, Patrick Pasquet

**Affiliations:** 1 CNRS, UMI 3189 «Environnement, Santé, Société», Faculté de Médecine Secteur Nord, Marseille, France; 2 CNRS, UMR 7206 «Eco-anthropologie et Ethnobiologie», Musée de l’Homme, Muséum National d’Histoire Naturelle, Paris, France; 3 MRC/Wits Developmental Pathways for Health Research Unit, Department of Paediatrics, Faculty of Health Sciences, University of the Witwatersrand, Johannesburg, South Africa; Newcastle University, UNITED KINGDOM

## Abstract

**Background:**

The social valorisation of overweight in African populations could promote high-risk eating behaviours and therefore become a risk factor of obesity. However, existing scales to assess body image are usually not accurate enough to allow comparative studies of body weight perception in different African populations. This study aimed to develop and validate the Body Size Scale (BSS) to estimate African body weight perception.

**Methods:**

Anthropometric measures of 80 Cameroonians and 81 Senegalese were used to evaluate three criteria of adiposity: body mass index (BMI), overall percentage of fat, and endomorphy (fat component of the somatotype). To develop the BSS, the participants were photographed in full face and profile positions. Models were selected for their representativeness of the wide variability in adiposity with a progressive increase along the scale. Then, for the validation protocol, participants self-administered the BSS to assess self-perceived current body size (CBS), desired body size (DBS) and provide a “body self-satisfaction index.” This protocol included construct validity, test-retest reliability and convergent validity and was carried out with three independent samples of respectively 201, 103 and 1115 Cameroonians.

**Results:**

The BSS comprises two sex-specific scales of photos of 9 models each, and ordered by increasing adiposity. Most participants were able to correctly order the BSS by increasing adiposity, using three different words to define body size. Test-retest reliability was consistent in estimating CBS, DBS and the “body self-satisfaction index.” The CBS was highly correlated to the objective BMI, and two different indexes assessed with the BSS were consistent with declarations obtained in interviews.

**Conclusion:**

The BSS is the first scale with photos of real African models taken in both full face and profile and representing a wide and representative variability in adiposity. The validation protocol proved its reliability for estimating body weight perception in Africans.

## Introduction

Over the past few decades the worldwide prevalence of obesity has dramatically increased and has become one of the most important public health issues [1]. Among other factors, urbanisation has been consistently associated with the obesity epidemic [1]. Nutritional transition exposes urban populations to a high-calorie diet, a low level of physical activity and sedentary behaviour, all risk factors in overweight and obesity [1]. Beyond weight status, measured by the body mass index (BMI), it has been shown that a high adiposity level (excess fat mass) increases risk of morbidity, especially of cardiovascular diseases [2]. Studies in Western countries have also highlighted that overweight weight and obesity are positively associated to mental disorders such as eating disorders and body dysmorphic disorders [3]. As a result, populations living in Western countries are faced with an ambivalent situation, where the prevalence of overweight and obesity is increasing while slimness is predominantly the desired body shape [4]. Given current urbanization rates, researchers have begun examining this ambivalence, especially in Africa [5].

Body weight perception in Black populations has been assessed in both Africa and Western countries [6, 7]. In both areas, studies have shown that the sociocultural valorisation of adiposity is a risk factor for obesity in Black populations [8–11]. Another issue tackled in the literature is the relationship between eating disorders, body dysmorphic disorders, and body weight perception among Black adolescents and adults living in urban areas of both developing and developed countries [12–14]. The results suggest an influence, through the media, of the modern conception of the ideal body size on body weight perception and on eating behaviours, especially in young adults [15]. Therefore, the literature highlights the necessity of understanding the changes in body weight perceptions to prevent high-risk eating behaviours and the incidence of obesity and associated diseases.

However, methodological issues limit our understanding of the relationship between body weight perception and biometric body size in African populations. So far, body weight perception has been assessed through qualitative and quantitative methods, including interviews [16], focus group [17], questionnaires [18], and figural stimuli representing different body sizes [19]. Two types of figural stimuli, drawings and silhouette scales, are preferentially used because of their ability to visually depict various human body sizes. However, these tools have limitations. Firstly, the drawings may be approximate since they are based on body size estimates and not on *actual* human shapes, potentially leading to misperceptions of body size in respondents [20–22]. Secondly, existing African scales are usually based on White Caucasian populations, and don’t fully cover the wide variability of phenotypes of Black populations, especially in morphology and skin colour [23, 24]. Thirdly, drawn scales are not developed using objective anthropometrical measures, limiting their use for comparing body weight perceptions to health outcomes [7, 21, 25–27]. In order to overcome these limitations, we designed the Body Image Scales (BIS) representing 6 real Cameroonian models selected according to their BMI [28]. Adapted to its ecological context, the BIS covers the variability of BMI of the Cameroonian population, but its ability to cover the adiposity variability is limited both by the number of models and the sole use of the BMI criterion. Additionally, the BIS contains only the front-view as most of body scales, whereas a recent study demonstrated the relevance of using both front and side-views in body image assessment [29].

The aim of the present study was to develop and validate the Body Size Scale (BSS): a new scale of photos of real models, more representative of African phenotypes with regard to body size, and taking into account more measures of adiposity, in order to specifically assess body weight perception with the aim to relating it to health outcomes.

## Methods

### Body Size Scale development

#### Targeted populations

Subjects were recruited in the Niger–Congo region, the largest ethno-linguistic area of Africa that spreads from Western to Southern Africa. In this area, populations were selected among the Bantoid (Western African ancestry group) and the Bantu (Central African ancestry group) populations, known as linguistically distinct [30], differing in macroscopic phenotypes in relation to their ecological context (lanky Western Bantoid in Sahel *vs*. stocky Bantu in the equatorial forest) [31], and in genotypes [32]. For that reason, the Bantoid were selected in Senegal and the Bantu in Cameroon.

#### Sampling

161 subjects (79 females) were sampled over a 2-month study in working-class neighbourhoods of Yaoundé (Cité Verte) and Dakar (Pikine). The participants in the development of the BSS were recruited as part of a neighbourhood health survey conducted by our team, where useful health information (BMI, glycaemia and blood pressure) was provided. Since the BSS focused on individuals of reproductive age, children, adolescents and elders only participated in the health survey and were not invited to take part in the BSS development. Pregnant women were also not invited since the body sharply changes during such a period.

#### Photos

In both cities, we rented a room in a house for one month. We installed our mobile photographic booth comprising a Canon EOS 450D camera (Tokyo, Japan) and an 18–55 mm lens, a tripod (set at a height of 1.0 m), a blue background (at a distance of 4 m from the camera), black skin-tight sportswear clothes. Through the network of our two informants in the field, we invited the neighbourhood residents to participate, only wearing the provided clothes (without jewellery or other distinctive objects). The participants stood on marks on the floor at 3.6 m from the camera, feet apart (0.5 m), arms wide apart from the torso (approximately 45°) and the palms of the hands facing outward. With the camera’s maximum image dimension (4272x2848 pixels), we photographed the participants both full face and in profile (left side of the body) to take into account body size variability on both the mediolateral and the antero-posterior axes. Indeed, this latter axis is usually not represented in existing body scales even though its potential contribution to body image studies has been raised [29, 33]. The procedure was similar in both cities, conducted by the first author, in collaboration with the fourth author and two informants.

#### Anthropometric measurements

We measured participants using standardized procedures to evaluate their BMI, body fat percentage, body fat distribution and somatotype [34]. Weight was measured to the nearest 0.1 kg, using a digital beam scale (Tanita, Tokyo, Japan). Height was measured to the nearest mm using a portable stadiometer (Siber-Hegner, Zurich, Switzerland). In standing position, hip, waist, upper arm (flexed and tensed) and calf circumferences were measured to the nearest mm using a tape measure [34]. Skinfold thickness (biceps, triceps, suprailiac, supraspinal, subscapular and medial calf) were measured to the nearest mm using a Harpenden skinfold caliper (Holtain Ltd., Crymych, UK). Two bone breadths (biepicondylar humerus and femur) were measured using a Mitutoyo Dial Caliper (Mitutoyo America, Aurora, Illinois, USA) [35]. We calculated BMI as weight (in kg) divided by the square of height (in m) and waist to hip ratio (WHR). We estimated fat mass (percentage of fat) by summing biceps, triceps, suprailiac and subscapular skinfolds [35]. We used Somatotype 1.0 software (MER Goulding Software Development, Geeveston, Australia) to evaluate each individual somatotype profile from ten anthropometric measures (height, weight, tricipital, subscapular, suprasinal and medial calf skinfolds, biepicondylar humerus and femur breadths, arm (flexed and tensed) and calf circumferences) [36]. The ternary components of somatotype (mesomorphy, ectomorphy and endomorphy) were plotted to represent participants’ index of musculature, slimness and fat, respectively.

#### Selection of models

Nine participants per sex were selected as models for the BSS after we carefully ensured both representative anthropometric variability and progressive adiposity gain along the scale. In our sample, the BMI frequency distribution led us to select the following number of models per BMI category: 1 in underweight (BMI ≤ 18.5 kg/m^2^), 3 in normal weight (18.5 < BMI ≤ 24.9 kg/m^2^); 2 in overweight (25.0 < BMI ≤ 29.9 kg/m^2^) and 1 in each class of obesity level as defined by the WHO (30.0 < BMI ≤ 34.9 kg/m^2^, 35.0 < BMI ≤ 39.9 kg/m^2^, and ≥ 40 kg/m^2^). We selected 3 models for the normal weight category and 2 for the overweight category because these categories are the most common in human populations. In addition, the models were selected by taking into account variability in body fat percentage and in endomorphy (fat component of the somatotype) in our sample. Adobe Photoshop CS software (Adobe Systems Inc, San Jose, CA) was used to edit the photos, mask the faces, remove the shadows and color the background in white.

### Body Size Scale validation

#### Population

In both urban and rural areas of Cameroon (Yaoundé, Bafoussam and Metet regions), we carried out a validation protocol following the standard procedure [37]. Households were randomly chosen by going door to door to every third house. Among adults of each selected household, we recruited one female and/or one male, and obtained their agreement to participate.

#### Body Size Scale use

For administering the BSS, the photos were randomly numbered from 1 to 9 and shuffled before asking the participants to sort the photos in ascending order of adiposity, without providing any information as to the anthropometric data associated with the models [37]. The male scale was given to males, and the female scale to females. In French, we evaluated the participant’s current body size (CBS) by asking “Peux-tu montrer l’image qui te ressemble le plus?” [Can you point out the model who looks like you the most?]. We evaluated the desired body size (DBS) by asking “Peux-tu montrer l’image à laquelle tu veux ressembler le plus?” [Can you point out the individual that you most want to look like?]. The CBS and DBS scores were defined as the number of the photo that the participants pointed out. We calculated the “desire to gain weight index” by subtracting the CBS from the DBS (DBS-CBS): a positive value indicates the desire to gain weight; a negative value, the desire to lose weight. We calculated the “body self-satisfaction index” as the absolute value of the “desire to gain weight index”: the higher the index value, the lower the body self-satisfaction [38].

#### Construct validity

To evaluate the construct validity of the BSS, we checked whether participants were able to order the BSS models by increasing adiposity along the scale [39]. We recruited 101 adults (45 females, 56 males) in rural Cameroon and 102 adults (51 females, 51 males) in urban Cameroon. Firstly, the participants were asked to re-arrange the photos without any specific instructions, to assess whether they had unconsciously integrated the progressive adiposity scale of the BSS. Then, we asked them to re-arrange the photos in ascending order of adiposity, using three words identified in a previous article as covering different local representations of body size: “gros” [big], “gras” [fat] and “corpulent” [stout], from the smallest to the largest size [28].

#### Test-retest reliability

To evaluate the reliability of assessing the CBS, the DBS, the “desire to gain weight index”, and the “body self-satisfaction index”, we administered the BSS as previously described twice (2 weeks apart) with 105 participants living in two Cameroonian cities, Bafoussam and Metet.

#### Convergent validity

To assess the convergent validity, we recruited 1116 adult Cameroonians living in Bafoussam, Metet and Yaoundé (599 females, 491 from rural areas). The aim was to compare the results from the BSS to information obtained by both objective anthropometric measures and an interview-administered questionnaire, as done in previous studies [20, 40]. We measured participants’ weight and height, and we derived the BMI, and overweight and obesity statuses. The following question was asked by questionnaire: “Voulez-vous perdre du poids, prendre du poids ou garder votre poids actuel?” [“Do you want to lose weight, gain weight or keep the weight you have now?”]).

### Statistics

The main characteristics of the three samples were described with means (± Standard Deviation, SD) and percentages.

In the sample used for the design of the BSS, we described the anthropometric variables through means (± SD) and compared them depending on the country of origin (Senegal *vs*. Cameroon) using analysis of variance. The comparisons of variables concerning body fat were adjusted for age. The somatotype analysis provided the somatotype status of each subject (endomorph, mesomorph or ectomorph). Frequency comparisons were then carried out to test the differences in somatotype status in regard to the country of origin, by using Fisher’s exact test. In the sample assembled for assessing construct validity, we calculated Cohen’s simple and weighted Kappas to compare agreement between the right ranking and the rankings established by the participants (no instructions, big, fat and stout). In brief, the simple kappa indicates whether the participant has or has not placed each photo in its expected position (right or wrong answer), while the weighted kappa takes into account the degree of error when wrong. For example, if the 4^th^ photo is placed in the 5^th^ position instead of the 4^th^, the simple kappa considers the answer wrong and the weighted kappa ascribes a lower degree of error than if the 4^th^ photo was placed in the 8^th^ position. The analysis was carried out separately depending on the sex and the living area in Cameroon.

The test-retest reliability for assessing CBS, DBS and a “desire to gain weight index” was evaluated using Spearman’s correlations, Cohen’s simple and weighted Kappas.

For convergent validity, we first assessed concurrent validity by estimating the Spearman’s correlation between the CBS and the objective BMI based on anthropometric measurements. Then, the ability of the CBS to predict overweight (yes *vs*. no) and obesity (yes *vs*. no) was assessed by estimating sensitivity (true positive rate) and specificity (true negative rate). This analysis was carried out separately for males and females. Finally, we assessed the ability of the BSS to predict the “desire to gain weight index”, and the ability of the “body self-satisfaction index” to predict these same indexes as obtained through interview-administered questionnaire elicitation, as previously carried out for validation studies of other body scales [41–43].

Statistical analyses were performed using SAS 9.3 software (SAS Institute, Cary, NC).

### Ethical standards disclosure

This study was conducted according to the guidelines laid down in the Helsinki Declaration and all procedures were approved by the Institutional Ethics Committee of the Institute of Medical Research and Medicinal Plant Studies of Cameroon. Verbal informed consent was obtained from all subjects. Verbal consent was witnessed and formally recorded.

## Results

### Development of the Body Size Scale

#### Anthropometric characteristics

**Fig 1:**
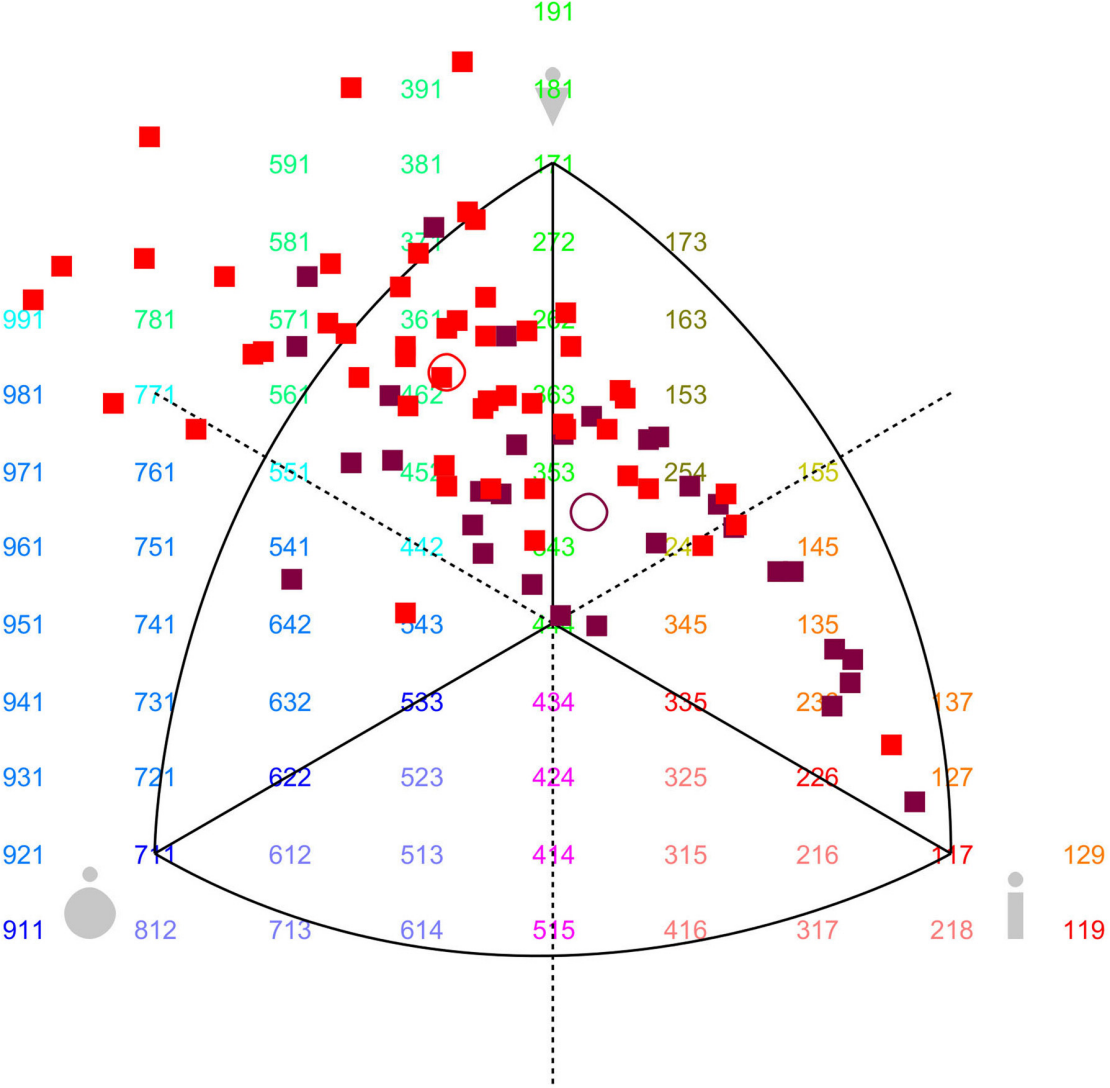
Somatotype profiles of male candidates as models for the BSS. In somatocharts, mesomorphy is represented by the vertical axis pointing upward; endomorphy by the horizontal axis pointing to the left; and ectomorphy by the horizontal axis pointing to the right. The red squares represent Cameroonian males and the purple ones Senegalese males. The red circle indicates the mean profile for Cameroonians and the purple one the mean profile for Senegalese.

**Fig 2:**
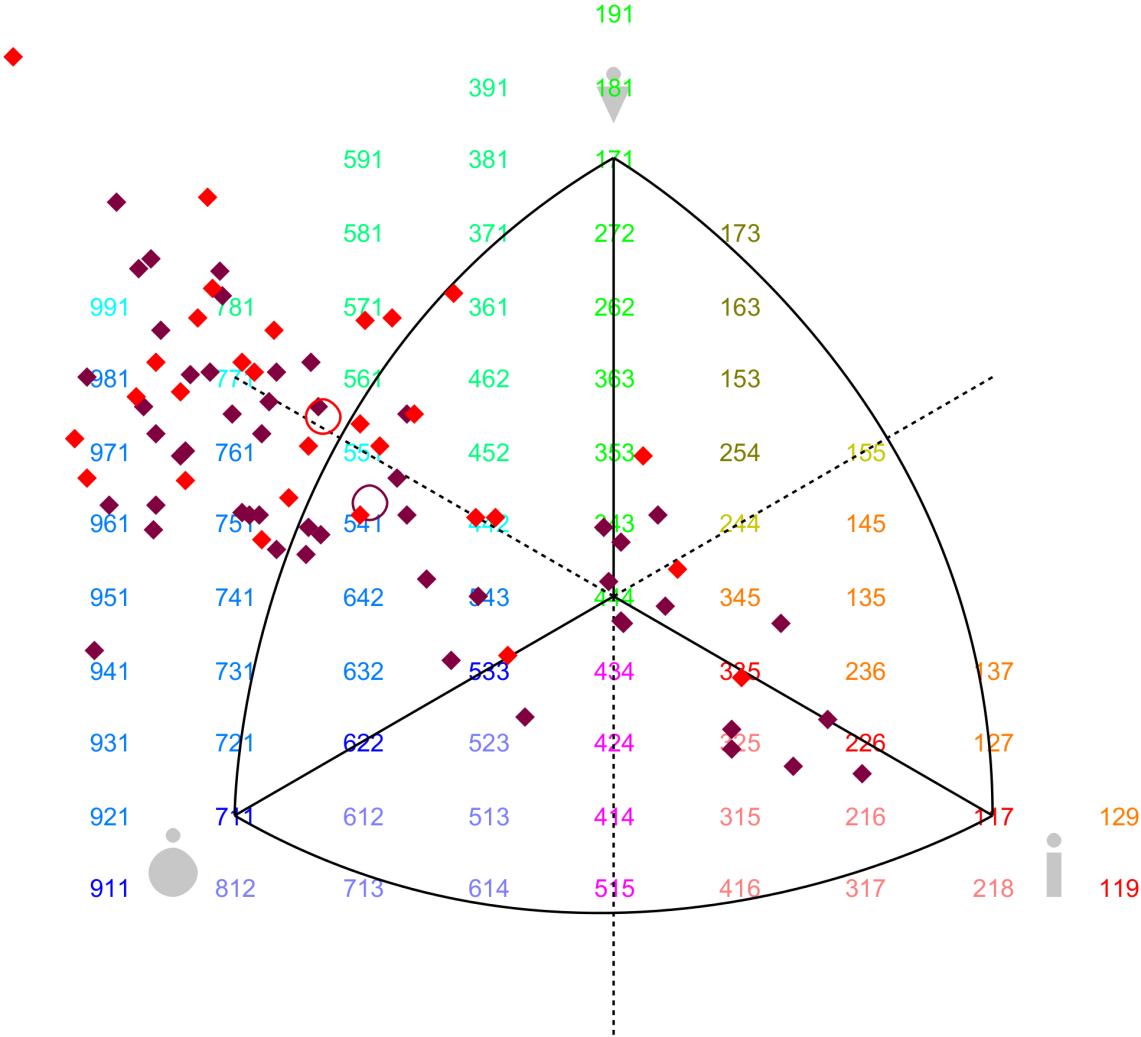
Somatotype profiles of female candidates as models for the BSS. In somatocharts, mesomorphy is represented by the vertical axis pointing upward; endomorphy by the horizontal axis pointing to the left; and ectomorphy by the horizontal axis pointing to the right. The red diamonds represent Cameroonian females and the purple ones Senegalese females. The red circle indicates the mean profile of Cameroonians and the purple one the mean profile of Senegalese.

**S1 Table** shows the anthropometric characteristics of the screened participants in the BSS development. In males, the Senegalese were 6 cm taller (p < 0.001) and had lower BMI (p < 0.01) and waist circumference (p < 0.001) than Cameroonians. In females, the Senegalese were 5 cm taller (p < 0.001), and had a lower BMI than Cameroonians (p < 0.05). Overall, Senegalese had a lower degree of mesomorphy (p < 0.001 for males, p < 0.05 for females) and a higher degree of ectomorphy (p < 0.001 for males, p < 0.01 for females). These results on the participants’ somatotypes are summed up by the somatocharts in **Figs 1**and **2**, for males and females respectively. The Cameroonian males were more likely to be mesomorph or endomorph, while Senegalese males were more likely to be mesomorph or ectomorph (p < 0.001). In females, differences were less pronounced since both Cameroonians and Senegalese were situated between endomorphy and mesomorphy, despite a subgroup shifted to the central and ectomorph region, especially among Senegalese females (p < 0.05).

#### The Body Size Scale

**Fig 3:**
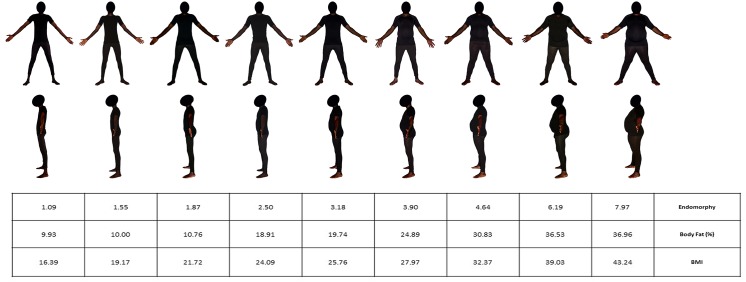
Male Body Size Scale. The scale shows 9 Black male models from front and left side-views, and ordered by increasing adiposity. The table gives the levels of endomorphy component, body fat percentage and body mass index corresponding to each model.

**Fig 4:**
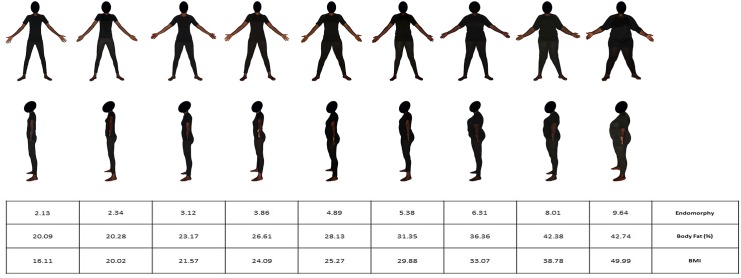
Female Body Size Scale. The scale shows 9 Black female models from front and left side-views, and ordered by increasing adiposity. The table gives the levels of endomorphy component, body fat percentage and body mass index corresponding to each model.

**Figs 3 and 4**show the photos of the 9 males and the 9 females selected as models for the BSS, together with their corresponding anthropometric data. For both scales, the models were ranked by increasing adiposity, as assessed by three anthropometric criteria: endomorphy, body fat percentage and BMI.

### Body Size Scale validation

A total of 201, 103 and 1115 Cameroonians were enrolled to respectively assess construct validity, test-retest reliability and convergent validity. Descriptive characteristics of these three independent samples are described in **Table 1**.

**Table 1:** Descriptive characteristics of the samples of population enrolled for the Body Size Scale validation protocol.

	Construct validity	SD	Test-retest reliability	SD	Convergent validity	SD
Sample size, *n*	201		103		1115	
Age, *y*	32.9	*14*.*5*	40.6	*14*.*8*	38.3	*13*.*7*
Sex, *% males*	52.7		45.7		47.4	
Area, *% rural*	49.8		100.0		43.9	

Abbreviation: SD, Standard Deviation

**Table 2**presents the results of the construct validity for the 201 participants from rural and urban areas. Males were more likely than females to set the BSS in the right order, regardless of the instructions given to participants. Except for in spontaneous order, all kappa coefficients were higher than 0.70 for males, which indicates good to excellent agreement between the participants’ and the expected rankings. However, males from rural areas consistently had lower kappa coefficients than those from urban areas. For females, the kappa coefficients were moderate to good. Compared to the females from urban areas, females from rural areas were more likely to spontaneously place the photos in the right order (without instructions) and to order the photos from the least big to the biggest. With regard to fatness and stoutness, rural and urban females had comparable kappa coefficients.

**Table 2:** κ and weighted κ corresponding to the construct validity of the Body Size Scale, according to sex and living area of the participants.

	Spontaneous order		less big to the biggest		less fat to the fattest		less stout to the stoutest	
	*κ*	*weighted κ*	*κ*	*weighted κ*	*κ*	*weighted κ*	*κ*	*weighted κ*
**Males**								
Rural area (n = 56)	0.60	0.66	0.72	0.76	0.73	0.77	0.79	0.81
Urban area (n = 51)	0.66	0.73	0.83	0.85	0.85	0.87	0.84	0.87
**Females**								
Rural area (n = 45)	0.51	0.58	0.70	0.74	0.60	0.64	0.68	0.71
Urban area (n = 51)	0.45	0.52	0.63	0.67	0.59	0.64	0.66	0.69

The results of test-retest reliability among the 103 participants are shown in **Table 3**. Correlations between the first and second assessments of Current Body Size (CBS), Desired Body Size (DBS) and the “body self-satisfaction index” were calculated for males and females. In both sexes, the correlation coefficients were found to be close to 0.60 and the weighted kappa coefficients were between 0.43 and 0.47 (moderate agreement between the first time tested and the second). Only the correlation coefficient and the weighted kappa coefficient were lower for the DBS in males (ρ = 0.39 and weighted κ = 0.27).

**Table 3:** Spearman’s correlation and Cohen’s simple and weighted kappas corresponding to the test-retest reliability of the Body Size Scale to assess current body size, desired body size and desire to gain weight in Cameroonians males and females.

	*ρ*	*κ*	*weighted κ*
**Males (n = 48)**			
CBS	0.60***	0.27	0.47
DBS	0.39**	0.14	0.27
Desire to gain weight index	0.53***		
**Females (n = 57)**			
CBS	0.59***	0.27	0.48
DBS	0.60***	0.27	0.43
Desire to gain weight index	0.55***		

Abbreviations: CBS, Current Body Size; DBS, Desired Body Size. P values of Spearman’s correlations: ** < 0.01

*** < 0.001.

Concurrent validity was assessed by calculating the correlation between CBS and BMI. Spearman’s coefficients were 0.72 for males and 0.59 for females (both p < 0.001). **Table 4**shows sex-specific sensitivity and specificity of the BSS regarding predictions as to overweight and obesity status, desire to gain weight and body self-satisfaction, assessed based on the sample of 1104 Cameroonians. As an example, 76% and 90% of overweight males and females (objective BMI > 25 kg/m^2^) were correctly identified as overweight (sensitivity) by the CBS (assessed using the BSS). The specificity was slightly lower, with 72% and 64% of the non-overweight (objective BMI < 25 kg/m^2^) males and females identified as non-overweight (specificity) by the CBS. Overall, all values of sensitivity and specificity were higher than 70%, except sensitivity to obesity in males (51%), and specificity of overweight in females (64%).

**Table 4:** Values of sensitivity and specificity of the Body Size Scale to identify overweight and obesity in both sexes, and the body weight self-satisfaction and the desire to gain weight in the overall sample.

	Sensitivity	Specificity
**Males (n = 525)**		
Overweight	76%	72%
Obesity	51%	93%
**Females (n = 579)**		
Overweight	90%	64%
Obesity	84%	85%
**Overall sample (n = 1104)**		
Desire to gain weight	78%	95%
Satisfaction	85%	90%

## Discussion

Our study presented the development and validation of the Body Size Scale (BSS), a sex-specific scale to assess body weight perception in African populations. It consists of two subscales of 9 photos of real Black models differing in their adiposity level, defined using three adiposity criteria: BMI, fat mass (skinfold thicknesses), and somatotype endomorphy component. A validation protocol was carried out in Cameroon and covered construct validity, test-retest reliability and convergent validity. Overall, this protocol gave moderate to excellent results concerning reliability, accuracy and precision of the BSS in Cameroonians.

The first strength of the BSS is that it overcomes major methodological issues by representing real Black models of each sex, and using both front and side views, as recently suggested [29]. So far, most existing body size scales aimed at assessing individual body weight perception use silhouettes or drawings [20, 21, 44]. Only a few used photos of real models [39, 40], especially in Africa [45]. Yet, the use of non-Black models or drawings could lead to misestimating body weight perception in Black participants. Moreover, some scales do not represent both sexes [21, 22, 40, 45]. Last, drawings or models are usually represented from the front [39, 40, 45], sometimes three-quarters [20] or in profile [22], but rarely from both front and profile [46]. This issue is critical since a profile view provides a representation of abdominal obesity [29]. Only computerized three-dimensional body image could constitute an alternative. In general, the method consists of asking participants to press a button to select their CBS or DBS on the screen with specific software [47]. Nevertheless, carrying a computer into the field to create in situ a digital simulation of body variation may not be convenient in some cases. The use of three-dimensional computer-generated images can save having to bring a computer to the field but this method remains less realistic than actual human photographs. The second strength of the BSS is the wider variability of African body size phenotypes, due to a higher number of models on the scale (9) and the use of 3 different adiposity criteria (BMI, fat mass and endomorphy) displaying high anthropometrical variation since Cameroonians and Senegalese have contrasted somatotype statuses. Other anthropometric criteria were not considered in the development of the BSS, including waist-to-hip ratio and the two other components of somatotypes (ectomorphy and mesomorphy). Indeed, our primary aim was to depict body size rather than body shape (i.e. shoulder and buttocks width) [48] in order to study body weight perception related to health outcomes due to adiposity.

The validation protocol provided evidence of the reliability and accuracy of using the BSS in Cameroonian populations. Firstly, the participants’ ability to correctly order the models by increasing adiposity based on three different words to define body weight was substantial to excellent (construct validity) since the weighted kappas were between 0.52 and 0.87. Second, test-retest reliability carried out 2 weeks apart were moderately good with correlation coefficients between 0.39 and 0.60 for CBS, DBS and the “desire to gain weight index”. Third, concurrent validity was good, since the correlation coefficients between CBS and BMI were 0.72 for males and 0.59 for females. The lower correlation for females could be explained by the fact that fat mass is scattered over several areas of the female body (legs, abdominal and gluteal regions), whereas in males the fat mass is more localised in the abdominal region [49]. Fourth, the assessment of convergent validity gave relatively high predictive values for participants’ overweight and obesity status, declared desire to gain weight and declared satisfaction in body weight. Overall, the reliability and the accuracy of the BSS were similar to other existing body size scales [50].

The main limitation of the BSS is that it was validated in Cameroon only. Even if body scales have been used with populations other than those for which they were initially developed [23, 51], the BSS may need to be validated for other African populations elsewhere in Africa or living in Western countries. The second limitation is that the BSS was not developed to evaluate aesthetic aspects since it is adapted to the assessment of body weight perception in relation to metabolic health outcomes through adiposity. Indeed, the study of aesthetic aspects and of sexual attractiveness would need to design a body shape scale taking into account other specific anthropometric criteria, such as WHR and the ectomorphy and mesomorphy components of somatotype [52]. In the near future, it will be possible to develop a specific body shape scale by integrating in its design specific anthropometric criteria such as WHR and the ectomorphy and mesomorphy components of somatotype related to sexual attractiveness [53].

## Conclusion

To conclude, the BSS is a reliable tool for assessing body weight perception in African populations. It enables characterization of the relationship between the social valorisation of overweight and obesity and associated metabolic diseases, by identifying potential misperceptions of adiposity levels. The BSS may also be used in comparative studies to assess differences in perception of overweight and obesity in native Africans and people of African descent living outside of Africa. Finally, in the context of nutritional transition and the obesity epidemic, the BSS may be used to estimate to what extent the social valorisation of overweight represents a risk factor for obesity and associated metabolic diseases [8, 10].

## Supporting Information

S1 Table Comparison of anthropometric characteristics between Cameroonians and Senegalese in both males and females for the development of the Body Size Scale. ^a^Adjusted for age. SD, Standard Deviation. Tests are analysis of variance between Cameroonians and Senegalese: NS, non-significant, *p<0.05, **p<0.01, ***p<0.001.
(DOCX)

## References

[1] Popkin BM, Adair LS, Ng SW. Global nutrition transition and the pandemic of obesity in developing countries. Nutr Rev. 2012;70(1):3–21. 10.1111/j.1753-4887.2011.00456.x 22221213 PMC3257829

[2] Grundy SM. Obesity, metabolic syndrome, and cardiovascular disease. J Clin Endocrinol Metab. 2004;89(6):2595–600. 10.1210/jc.2004-0372 . 15181029

[3] Wadden TA, Stunkard AJ. Social and psychological consequences of obesity. Ann Intern Med. 1985;103(6 (Pt 2)):1062–7. 10.7326/0003-4819-103-6-1062 . 4062126

[4] Dave D, Rashad I. Overweight status, self-perception, and suicidal behaviors among adolescents. Soc Sci Med. 2009;68(9):1685–91. 10.1016/j.socscimed.2009.02.015 19297063

[5] Véron J. La moitié de la population mondiale vit en ville. Population & Sociétés. 2007;435:1–4.

[6] Gitau TM, Micklesfield LK, Pettifor JM, Norris SA. Ethnic differences in eating attitudes, body image and self-esteem among adolescent females living in urban South Africa. Journal of Psychiatry. 2014;17:468–74. doi: 10.4172/Psychiatry.1000101.

[7] Becker DM, Yanek LR, Koffman DM, Bronner YC. Body image preferences among urban African Americans and whites from low income communities. Ethn Dis. 1999;9(3):377–86. 10600060

[8] Cohen E, Boetsch G, Palstra FP, Pasquet P. Social valorisation of stoutness as a determinant of obesity in the context of nutritional transition in Cameroon: the Bamileke case. Soc Sci Med. 2013;96:24–32. 10.1016/j.socscimed.2013.07.004 . 24034948

[9] Puoane T, Fourie JM, Shapiro M, Rosling L, Tshaka NC, Oelefse A. 'Big is beautiful'-an exploration with urban black community health workers in a South African township. South Afr J Clin Nutr. 2005;18(1):6–15.

[10] Hendley Y, Zhao L, Coverson DL, Din-Dzietham R, Morris A, Quyyumi AA, et al Differences in weight perception among blacks and whites. J Womens Health (Larchmt). 2011;20(12):1805–11. 10.1089/jwh.2010.2262 21988528 PMC3236990

[11] Flynn KJ, Fitzgibbon M. Body images and obesity risk among Black females: A review of the literature. Ann Behav Med. 1998;20(1):13–24. 10.1007/bf02893804 9755347

[12] Gitau TM, Micklesfield LK, Pettifor JM, Norris SA. Eating attitudes, body image satisfaction and self-esteem of South African Black and White male adolescents and their perception of female body silhouettes. J Child Adolesc Ment Health. 2014;26(3):193–205. 10.2989/17280583.2014.901224 . 25533406

[13] Madowitz J. "Il faut manger": Eating Disorders in Cameroonian Women. Collection ISPI, editor2006. 266 p.

[14] Cachelin FM, Rebeck RM, Chung GH, Pelayo E. Does ethnicity influence body-size preference? A comparison of body image and body size. Obes Res. 2002;10(3):158–66. 10.1038/oby.2002.25 . 11886938

[15] Borzekowski D, Bayer AM. Body image and media use among adolescents. Adolesc Med Clin. 2005;16(2):289–313. 16111619 10.1016/j.admecli.2005.02.010

[16] Mvo Z. Perceptions of overweight African women about acceptable body size of women and children. Curationis. 1999;22(2):27–31. 10.4102/curationis.v22i2.719 11040616

[17] Puoane T, Tsolekile L, Steyn N. Perceptions about body image and sizes among black African girls living in Cape Town. Ethn Dis. 2010. 20178179

[18] Cash TF, Melnyk SE, Hrabosky JI. The assessment of body image investment: an extensive revision of the appearance schemas inventory. The International journal of eating disorders. 2004;35(3):305–16. 10.1002/eat.10264 . 15048946

[19] Rguibi M, Belahsen R. Body size preferences and sociocultural influences on attitudes towards obesity among Moroccan Sahraoui women. Body Image. 2006;3(4):395–400. 10.1016/j.bodyim.2006.07.007 . 18089243

[20] Williamson DA, Womble LG, Zucker NL, Reas DL, White MA, Blouin DC, et al Body image assessment for obesity (BIA-O): development of a new procedure. Int J Obes Relat Metab Disord. 2000;24(10):1326–32. 10.1038/sj.ijo.0801363 . 11093295

[21] Patt MR, Lane AE, Finney CP, Yanek LR, Becker DM. Body image assessment: comparison of figure rating scales among urban Black women. Ethn Dis. 2002;12(1):54–62. PubMed Central PMCID: PMC11913609 11913609

[22] Marlowe F, Apicella C, Reed D. Men's preferences for women's profile waist-to-hip ratio in two societies. Evol Hum Behav. 2005;26(6):458–68. 10.1016/j.evolhumbehav.2005.07.005

[23] Holdsworth M, Gartner A, Landais E, Maire B, Delpeuch F. Perceptions of healthy and desirable body size in urban Senegalese women. Int J Obes Relat Metab Disord. 2004;28(12):1561–8. 10.1038/sj.ijo.0802739 . 15278107

[24] Flynn K, Fitzgibbon M. Body image ideals of low-income African American mothers and their preadolescent daughters. J Youth Adolesc. 1996;25(5):615–30. 10.1007/bf01537357

[25] Pulvers KM, Lee RE, Kaur H, Mayo MS, Fitzgibbon ML, Jeffries SK, et al Development of a culturally relevant body image instrument among urban African Americans. Obes Res. 2004;12(10):1641–51. 10.1038/oby.2004.204 . 15536228

[26] Jumah NA, Duda RB. Comparison of the perception of ideal body images of Ghanaian men and women. Afr J Health Sci. 2008;14(1):54–60. 10.4314/ajhs.v14i1.30847

[27] McIza Z, Goedecke JH, Steyn NP, Charlton K, Puoane T, Meltzer S, et al Development and validation of instruments measuring body image and body weight dissatisfaction in South African mothers and their daughters. Public Health Nutr. 2007;8(05):509–19. 10.1079/phn2005814 16153332

[28] Cohen E, Pasquet P. Development of a new body image assessment scale in urban Cameroon: an anthropological approach. Ethn Dis. 2011;21(3):288–93. 21942160

[29] Cohen E, Ndao A, Boetsch G, Gueye L, Pasquet P, Holdsworth M, et al The contribution of the side-view in body image scales for public health: an example from two African populations. BMC Public Health. 2015;(In press). 10.1186/s12889-015-2511-x PMC4659201 26603149

[30] Blench R. Why Is Africa So Linguistically Undiverse? Exploring Substrates and Isolates. Mother Tongue. 2013;(XVIII).

[31] Froment A, Hiernaux J. Climate-associated anthropometric variation between populations of the Niger bend. Ann Hum Biol. 1984;11(3):189–200. 10.1080/03014468400007061 6742769

[32] Montano V, Ferri G, Marcari V, Batini C, Anyaele O, Destro-Bisol G, et al The Bantu expansion revisited: a new analysis of Y chromosome variation in Central Western Africa. Mol Ecol. 2011;20(13):2693–708. 10.1111/j.1365-294X.2011.05130.x . 21627702

[33] Swami V, Tovee MJ. The relative contribution of profile body shape and weight to judgements of women's physical attractiveness in Britain and Malaysia. Body Image. 2007;4(4):391–6. 10.1016/j.bodyim.2007.07.002 . 18089286

[34] Weiner JS, Lourie JA. Practical Human Biology. London: Academic Press; 1981. 439 p.

[35] Durnin JV, Womersley J. Body fat assessed from total body density and its estimation from skinfold thickness: measurements on 481 men and women aged from 16 to 72 years. Br J Nutr. 1974;32(1):77–97. 10.1079/BJN19740060 . 4843734

[36] Carter JL, Heath BH. Somatotyping: development and applications Melbourne, Australia: Cambridge University Press; 1990.

[37] Gardner RM, Friedman BN, Jackson NA. Methodological concerns when using silhouettes to measure body image. Percept Mot Skills. 1998;86(2):387–95. 10.2466/pms.1998.86.2.387 . 9638738

[38] Williamson DA, Gleaves DH, Watkins PC, Schlundt DG. Validation of self-ideal body size discrepancy as a measure of body dissatisfaction. J Psychopathol Behav Assess. 1993;15(1):57–68. 10.1007/bf00964324

[39] Harris CV, Bradlyn AS, Coffman J, Gunel E, Cottrell L. BMI-based body size guides for women and men: development and validation of a novel pictorial method to assess weight-related concepts. Int J Obes (Lond). 2008;32(2):336–42. 10.1038/sj.ijo.0803704 . 17700580

[40] Swami V, Salem N, Furnham A, Tovée MJ. Initial examination of the validity and reliability of the female photographic figure rating scale for body image assessment. Pers Individ Dif. 2008;44(8):1752–61. 10.1016/j.paid.2008.02.002

[41] Maupin JN, Hruschka DJ. Assessing the accuracy of two proxy measures for BMI in a semi-rural, low-resource setting in Guatemala. BMC Public Health. 2014;14(1):973 10.1186/1471-2458-14-973 25238737 PMC4190281

[42] Pulvers K, Bachand J, Nollen N, Guo H, Ahluwalia JS. BMI-based norms for a culturally relevant body image scale among African Americans. Eating behaviors. 2013;14(4):437–40. 10.1016/j.eatbeh.2013.07.005 24183131 PMC3817499

[43] Lo W-S, Ho S-Y, Mak K-K, Lam T-H. The Use of Stunkard’s figure rating scale to identify underweight and overweight in Chinese adolescents. PloS one. 2012;7(11):e50017 10.1371/journal.pone.0050017 23189177 PMC3506537

[44] Stunkard AJ, Sørensen T, Schulsinger F. Use of the Danish Adoption Register for the study of obesity and thinness. Res Publ Assoc Res Nerv Ment Dis. 1983;60:115 PubMed Central PMCID: PMC6823524. 6823524

[45] Tovee MJ, Hancock PJ, Mahmoodi S, Singleton BR, Cornelissen PL. Human female attractiveness: waveform analysis of body shape. Proc Biol Sci. 2002;269(1506):2205–13. 10.1098/rspb.2002.2133 12427313 PMC1691155

[46] Bush HM, Williams RG, Lean ME, Anderson AS. Body image and weight consciousness among South Asian, Italian and general population women in Britain. Appetite. 2001;37(3):207–15. 10.1006/appe.2001.0424 . 11895321

[47] Stewart TM, Allen HR, Han H, Williamson DA. The development of the Body Morph Assessment version 2.0 (BMA 2.0): tests of reliability and validity. Body Image. 2009;6(2):67–74. 10.1016/j.bodyim.2009.01.006 19244002 PMC2743122

[48] Ritenbaugh C. Body size and shape: a dialogue of culture and biology. Medical anthropology. 1991;13(3):173–80. 10.1080/01459740.1991.9966047 . 1961101

[49] Karastergiou K, Smith SR, Greenberg AS, Fried SK. Sex differences in human adipose tissues—the biology of pear shape. Biol Sex Differ. 2012;3(1):13 10.1186/2042-6410-3-13 22651247 PMC3411490

[50] Gardner RM, Brown DL. Body image assessment: A review of figural drawing scales. Pers Individ Dif. 2010;48(2):107–11. 10.1016/j.paid.2009.08.017

[51] Swami V, Mada R, Tovee MJ. Weight discrepancy and body appreciation of Zimbabwean women in Zimbabwe and Britain. Body Image. 2012;9(4):559–62. 10.1016/j.bodyim.2012.05.006 . 22717762

[52] Stewart AD, Benson PJ, Michanikou EG, Tsiota DG, Narli MK. Body image perception, satisfaction and somatotype in male and female athletes and non-athletes: results using a novel morphing technique. J Sports Sci. 2003;21(10):815–23. 10.1080/0264041031000140338 . 14620025

[53] Dixson BJ, Dixson AF, Morgan B, Anderson MJ. Human physique and sexual attractiveness: sexual preferences of men and women in Bakossiland, Cameroon. Arch Sex Behav. 2007;36(3):369–75. 10.1007/s10508-006-9093-8 . 17136587

