# Supplementary material for: Development and Validation of the Body Size Scale for Assessing Body Weight Perception in African Populations
Source: PLoS One. 2015 Nov 4;10(11):e0138983. doi: 10.1371/journal.pone.0138983 (PMC4633130; doi:10.1371/journal.pone.0138983)
Supplement: S1 Table — aAdjusted for age. SD, Standard Deviation. Tests are analysis of variance between Cameroonians and Senegalese: NS, non-significant, *p<0.05, **p<0.01, ***p<0.001. (DOCX) [file pone.0138983.s001.docx]

|  | Males | | | | | Females | | | | |
| --- | --- | --- | --- | --- | --- | --- | --- | --- | --- | --- |
|  | Cameroonian | | Senegalese | |  | Cameroonian | | Senegalese | |  |
|  | Mean | *SD* | Mean | *SD* | *p* | Mean | *SD* | Mean | *SD* | *p* |
| *N* | 51 | | 31 | |  | 29 | | 50 | |  |
| Age (y) | 39.5 | 14.2 | 37.5 | 16.0 | NS | 35.3 | 12.0 | 39.9 | 13.4 | NS |
| Height (cm) | 170.8 | 6.2 | 176.9 | 6.2 | *** | 160.4 | 6.3 | 165.0 | 5.4 | *** |
| Weight (kg) | 77.6 | 15.0 | 71.9 | 15.0 | NS | 75.4 | 16.4 | 72.7 | 16.4 | NS |
| BMI^a^ (kg/m^2^) | 26.5 | 4.5 | 23 | 4.5 | ** | 29.4 | 6.0 | 26.6 | 5.94 | * |
| Waist circumference^a^ (cm) | 90.4 | 11.6 | 81.2 | 11.6 | *** | 95.9 | 14.2 | 91.9 | 14.2 | NS |
| Hip circumference^a^ (cm) | 99.2 | 9.7 | 95.4 | 9.7 | NS | 109.4 | 12.1 | 104.8 | 12.1 | NS |
| WHR^a^ | 0.9 | 0.1 | 0.8 | 0.1 | NS | 0.9 | 0.08 | 0.9 | 0.08 | NS |
| Body fat^a^ (%) | 19.9 | 6.0 | 18.6 | 6.0 | NS | 34.4 | 6.0 | 33.1 | 5.9 | NS |
| Biepicondylar humerus (cm) | 6.9 | 0.4 | 7.0 | 0.4 | NS | 6.2 | 0.3 | 6.3 | 0.3 | NS |
| Biepicondylar femur (cm) | 9.3 | 0.8 | 9.4 | 0.8 | NS | 8.9 | 0.6 | 8.9 | 0.6 | NS |
| Endomorphy^a^ | 3.3 | 1.4 | 2.7 | 1.4 | NS | 5.7 | 1.8 | 5.4 | 1.8 | NS |
| Mesomorphy^a^ | 5.8 | 1.4 | 4.4 | 1.4 | *** | 5.8 | 1.8 | 4.8 | 1.8 | * |
| Ectomorphy^a^ | 1.7 | 1.4 | 3.2 | 1.4 | *** | 0.7 | 1.4 | 1.8 | 1.4 | ** |
